# Analysis of Essential Isoprene Metabolic Pathway Proteins in *Variovorax* sp. Strain WS11

**DOI:** 10.1128/aem.02122-22

**Published:** 2023-02-22

**Authors:** Gregory D. Rix, Leanne P. Sims, Robin A. Dawson, Gordon Williamson, Yasmin Bryant, Andrew T. Crombie, J. Colin Murrell

**Affiliations:** a School of Environmental Science, University of East Anglia, Norwich, United Kingdom; University of Michigan-Ann Arbor

**Keywords:** climate change, isoprene, bioinformatics, protein purification

## Abstract

Isoprene monooxygenase (IsoMO, encoded by *isoABCDEF*) initiates the oxidation of the climate-active gas isoprene, with the genes *isoGHIJ* and *aldH* nearly always found adjacent to *isoABCDEF* in extant and metagenome-derived isoprene degraders. The roles of *isoGHIJ* and *aldH* are uncertain, although each is essential to isoprene degradation. We report here the characterization of these proteins from two model isoprene degraders, *Rhodococcus* sp. strain AD45 and *Variovorax* sp. strain WS11. The genes *isoHIJ* and *aldH* from *Variovorax* and *aldH* from *Rhodococcus* were expressed individually in Escherichia coli as maltose binding protein fusions to overcome issues of insolubility. The activity of two glutathione *S*-transferases from *Variovorax*, IsoI and IsoJ was assessed with model substrates, and the conversion of epoxyisoprene to the intermediate 1-hydroxy-2-glutathionyl-2-methyl-3-butene (HGMB) was demonstrated. The next step of the isoprene metabolic pathway of *Variovorax* is catalyzed by the dehydrogenase IsoH, resulting in the conversion of HGMB to 2-glutathionyl-2-methyl-3-butenoic acid (GMBA). The aldehyde dehydrogenases (AldH) from *Variovorax* and *Rhodococcus* were examined with a variety of aldehydes, with both exhibiting maximum activity with butanal. AldH significantly increased the rate of production of NADH when added to the IsoH-catalyzed conversion of HGMB to GMBA (via GMB), suggesting a synergistic role for AldH in the isoprene metabolic pathway. An *in silico* analysis of IsoG revealed that this protein, which is essential for isoprene metabolism in *Variovorax*, is an enzyme of the formyl CoA-transferase family and is predicted to catalyze the formation of a GMBA-CoA thioester as an intermediate in the isoprene oxidation pathway.

**IMPORTANCE** Isoprene is a climate-active gas, largely produced by trees, which is released from the biosphere in amounts equivalent to those of methane and all other volatile organic compounds combined. Bacteria found in many environments, including soils and on the surface of leaves of isoprene-producing trees, can grow on isoprene and thus may represent a significant biological sink for this globally significant volatile compound and remove isoprene before it escapes to the atmosphere, thus reducing its potency as a climate-active gas. The initial oxidation of isoprene by bacteria is mediated by isoprene monooxygenase encoded by the genes *isoABCDEF*. In isoprene-degrading bacteria, a second gene cluster, *isoGHIJ*, is also present, although the exact role in isoprene degradation by the proteins encoded by these genes is uncertain. This investigation sheds new light on the roles of these proteins in the isoprene oxidation pathway in two model isoprene-degrading bacteria of the genera *Rhodococcus* and *Variovorax.*

## INTRODUCTION

Isoprene (2-methyl-1,3-butadiene) is one of the most abundantly produced biogenic volatile organic compounds (BVOC), with global atmospheric emissions of ~500 teragrams (Tg year^−1^) (1), roughly equal to the global emissions of methane and also equal to emissions of all other nonmethane BVOCs combined (2). Isoprene is produced primarily by terrestrial plants such as trees, mosses, and ferns (3). Other sources include marine algae, which emit approximately 0.1 to 12 Tg year^−1^ (4, 5), some bacteria, fungi, and animals (6). Anthropogenic sources of isoprene include the industrial production of polyisoprene rubber, at an annual production of 0.8 Tg isoprene year^−1^, and vehicle emissions (7).

A landmark experiment by Cleveland and Yavitt identified soils as a significant sink for atmospheric isoprene, with early estimates of global isoprene consumption reaching 20.4 Tg year^−1^ (8). These and later experiments revealed that this isoprene sink was microbially driven and could offset global emissions by 4% or more (9–11). Since then, the diversity of isoprene-degrading communities has been investigated through a combination of culture-dependent and culture-independent techniques. Isoprene-degrading bacteria of the genera *Rhodococcus*, *Nocardia*, *Arthrobacter*, and *Alcaligenes* were isolated from soils (12, 13) and phyllosphere and soils of isoprene-emitting trees such as oil palm, willow, poplar, and oak (14–16). These studies demonstrate the diversity of Gram-positive and Gram-negative isoprene-degrading bacteria in the environment (2, 6, 17).

Stable-isotope probing (DNA-SIP) experiments showed that *Rhodococcus* spp. were abundant and active members of the isoprene-degrading soil community (15, 18, 19). The most well-characterized isoprene degrader was the Gram-positive *Rhodococcus* sp. strain AD45, isolated from freshwater sediment (20). More recently, members of the *Betaproteobacteria* have been identified as prominent members of the isoprene-degrading community in soil and phyllosphere associated with poplar and willow trees (14, 21). *Variovorax* sp. strain WS11, isolated from willow soil, is now one of the most well-characterized isoprene degraders (21–23).

**FIG 1:**
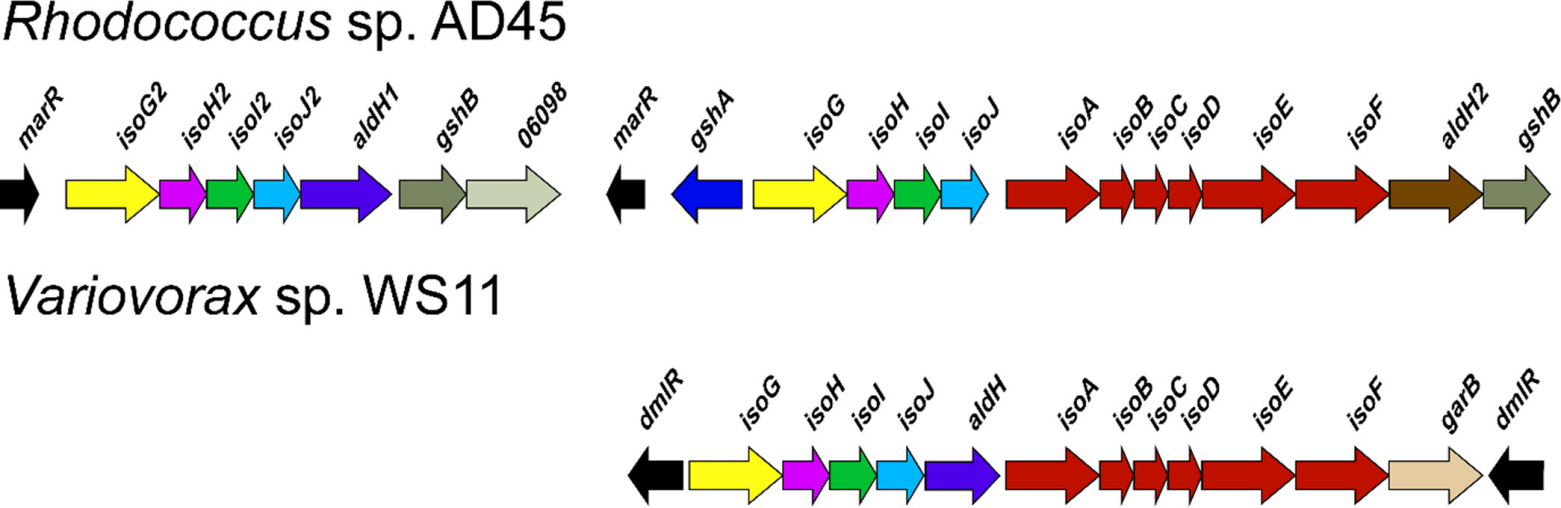
Comparison of the isoprene metabolic gene cluster organization of the most well-characterized isoprene degraders, *Rhodococcus* sp. AD45 and *Variovorax* sp. WS11. The isoprene cluster contain six genes (*isoABCDEF*) encoding an α_2_β_2_γ_2_ oxygenase (IsoABE), a reductase (IsoF), a Rieske-type ferredoxin (IsoC), and a coupling protein (IsoD) that form the isoprene monooxygenase (IsoMO). Five additional genes (*isoGHIJ* and *aldH*) (*Rhodococcus* sp. AD45 contains two copies of *isoGHIJ*) are located upstream of the monooxygenase genes *isoABCDEF* and encode a putative coenzyme A transferase, an NAD^+^-dependent dehydrogenase, two glutathione *S*-transferases (GSTs), and a putative dehydrogenase (AldH), respectively.

All isoprene degraders examined contain six genes (*isoABCDEF*) encoding an α_2_β_2_γ_2_ oxygenase (IsoABE), a reductase (IsoF), a Rieske-type ferredoxin (IsoC), and a coupling protein (IsoD) that form the isoprene monooxygenase (IsoMO) (24). Five additional genes (*isoGHIJ* and *aldH*), encoding a putative coenzyme A transferase, an NAD^+^-dependent dehydrogenase, two glutathione *S*-transferases (GSTs), and a putative dehydrogenase (AldH), respectively, are located upstream of the IsoMO-encoding genes (22, 25). This appears to be the typical organization for *iso* genes in all isoprene-degrading bacteria studied in detail, with the exception that in *Rhodococcus.* sp. AD45, *aldH* is located further upstream, adjacent to duplicate copies of *isoGHIJ* (Fig. 1). Recently, the genome of an isoprene-degrading *Alcaligenes* sp. was shown to lack a conventional *iso* metabolic gene cluster (26).

**FIG 2:**
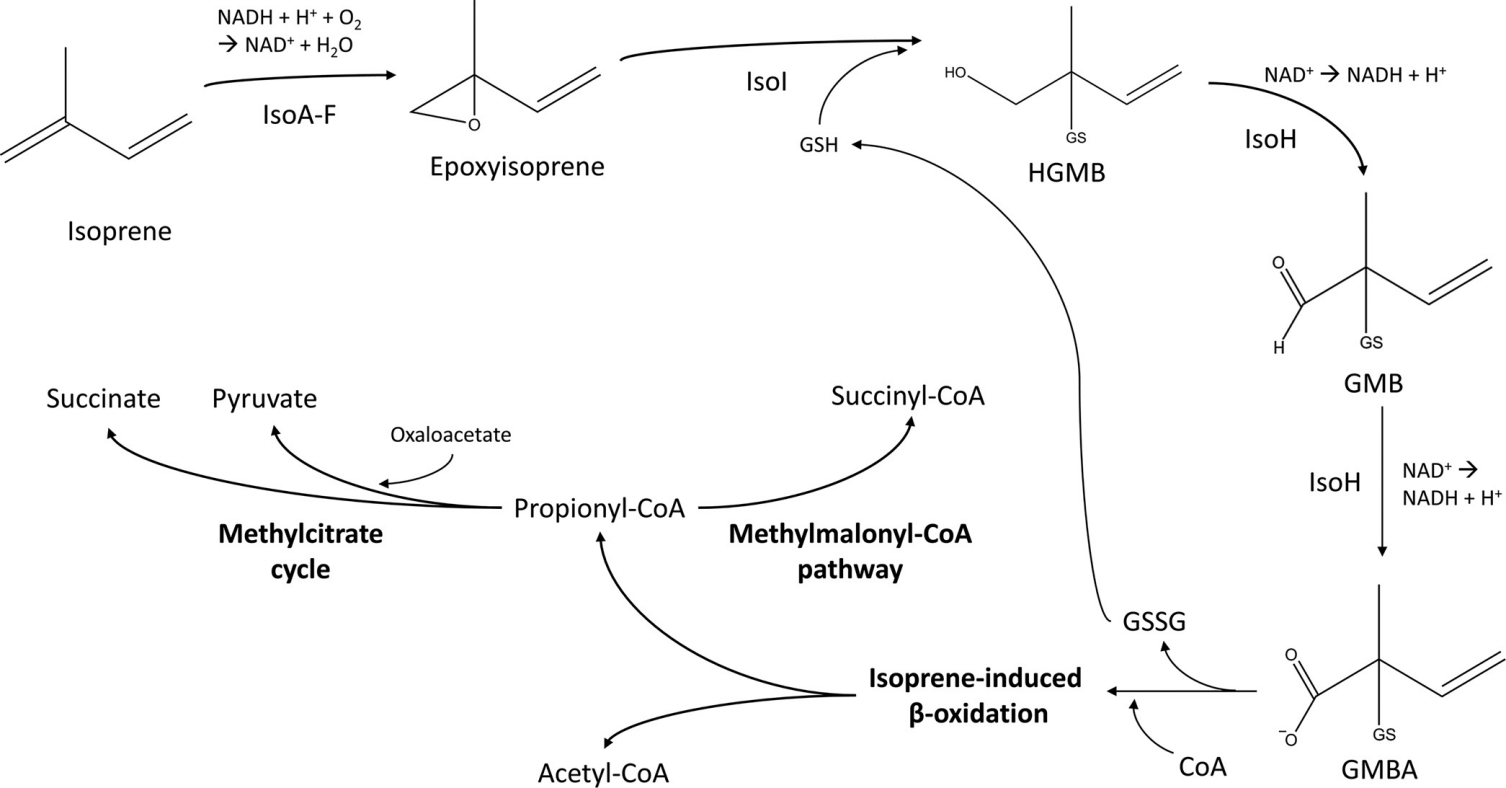
Isoprene metabolic pathway in the model bacterium *Rhodococcus* sp. AD45. HGMB, 1-hydroxy-2-glutathionyl-2-methyl-3-butene; GMB, 2-glutathionyl-2-methyl-3-butenal; GMBA, 2-glutathionyl-2-methyl-3-butenoic acid; SG, glutathione; GSH, reduced glutathione; X-CoA, donor (25). Adapted from 42.

The initial enzyme reactions mediating isoprene degradation in *Rhodococcus* sp. AD45 were described by van Hylckama Vlieg et al. (20, 25, 27). Briefly, isoprene is oxidized to epoxyisoprene by IsoMO (24). The epoxide is then detoxified by conjugation with glutathione, a low-molecular-mass thiol not typically found in Gram-positive bacteria (28, 29), forming 1-hydroxy-2-glutathionyl-2-methyl-3-butene (HGMB), a reaction catalyzed by IsoI. HGMB is converted to 2-glutathionyl-2-methyl-3-butenal (GMB) and then to 2-glutathionyl-2-methyl-3-butenoic acid (GMBA) by two successive NAD^+^-dependent oxidation reactions, catalyzed by IsoH. The subsequent steps have yet to be elucidated, but it is predicted that carbon from GMBA enters the central metabolism via a β-oxidative metabolic pathway (Fig. 2). Pathways of carbon assimilation from isoprene by *Variovorax* WS11 have recently been investigated using transcriptomics, proteomics, and mutagenesis approaches (23) and involves the conversion of GMBA to a CoA-thioester by IsoG and the removal of glutathione from the GMBA-CoA molecule by IsoJ.

There is still no clear role in the predicted isoprene metabolic pathway for AldH, despite AldH being essential for growth of *Variovorax* sp. WS11 on isoprene (23). A putative role for AldH was suggested by a study of an analogous pathway of styrene metabolism in which an AldH homologue may act alongside StyH in the conversion of (S)-1-phenyl-2-acetaldehyde glutathione to (S)-1-phenyl-2-acetic acid glutathione (30, 31). The equivalent reaction in the isoprene metabolic pathway is the oxidation of GMB to GMBA. However, if AldH does indeed catalyze the oxidation of GMB to GMBA, a reaction which is also catalyzed by IsoH (27), then it is unclear how the deletion of the *aldH* gene resulted in the total prevention of growth on isoprene in *Variovorax* sp. WS11 (23).

Glutathione *S*-transferases (GSTs), present in both eukaryotes and prokaryotes, detoxify a range of xenobiotics through conjugation with a glutathione molecule (32). Bacterial GSTs have been identified in gene clusters coding for aerobic degradation of aromatic compounds and epoxides (33, 34). Initial characterization of the GSTs IsoI and IsoJ was performed in *Rhodococcus* sp. AD45 (20, 25, 27). Recently, an analogous pathway of styrene metabolism was discovered in Gordonia rubripertincta CWB2 in which two GSTs, StyI and StyJ, having homology to IsoI and IsoJ, were involved in styrene degradation (31). In *Rhodococcus* sp. AD45 and *Variovorax* sp. WS11, the isoprene metabolic gene clusters, containing glutathione *S*-transferase-encoding genes (*isoI* and *isoJ*), are located on megaplasmids (22, 35), suggesting acquisition of glutathione usage by horizontal gene transfer. Therefore, comparison of these highly similar proteins utilized in the same metabolic pathway from both Gram-positive and Gram-negative isoprene degraders presents a unique opportunity to elucidate their properties.

Here, the *isoHIJ* and *aldH* genes from *Variovorax* sp. WS11 and *aldH* from *Rhodococcus* sp. AD45 (here prefixed with WS11- and AD45-, respectively) were expressed heterologously in Escherichia coli, and the resulting proteins were purified to facilitate biochemical characterizations. Using a combination of newly developed high-performance liquid chromatography (HPLC), gas chromatography-mass spectrometry (GC-MS), liquid chromatography-mass spectrometry (LC-MS), and spectroscopic assays, the production of intermediary metabolites in the isoprene metabolic pathway was examined, together with investigations into the roles of the essential proteins WS11-IsoG and WS11-IsoJ.

## RESULTS AND DISCUSSION

### Purification of enzymes from the isoprene metabolic pathway.

WS11-IsoH, WS11-IsoI, WS11-AldH, and AD45-AldH were purified as recombinant N-terminal His-tagged constructs with predicted molecular masses, including the His-tag, of 25.6, 27.9, 51.6, and 51.5 kDa, respectively. To overcome issues with solubility, WS11-IsoJ was expressed with an N-terminal maltose binding protein fusion, which helps to facilitate the proper folding and solubility of the recombinant protein. Specific activities for WS11-IsoI and WS11-IsoJ were determined using the model GST substrates, 1-chloro-2,4-dinitrobenzene (CDNB) and 1,2-dichloro-4-nitrobenzene (DCNB). No activity was detected with DCNB for either WS11-IsoI or WS11-IsoJ. The specific activities of WS11-IsoI and WS11-IsoJ were very low with CDNB, at 0.028 ± 0.009 and 0.023 ± 0.004 U mg^−1^, respectively. In comparison with previously published data for AD45-IsoI and AD45-IsoJ, both IsoJ proteins displayed similar activities, whereas WS11-IsoI exhibited CDNB activity while AD45-IsoI did not (25). More recently, the two GSTs encoded in the styrene degradation pathway, StyI and StyJ, having 44% and 48% amino acid sequence identity with WS11-IsoI and WS11-IsoJ, respectively, were analyzed using CDNB and DCNB. Similarly, StyI and StyJ showed no activity toward DCNB. With CDNB, WS11-IsoI displayed 177% higher activity than its homologue StyI, but WS11-IsoJ activity was only 6.6% that of StyJ. Confirmation that WS11-IsoJ functions as a glutathione *S*-transferase is consistent with previous predictions that IsoJ may catalyze the removal of glutathione from a GMBA-CoA thioester as glutathione-disulfide (23, 25). Although this role is yet to be ratified, deletion of the *isoJ* gene prevented growth of *Variovorax* sp. WS11 on isoprene (23), confirming that IsoJ is essential for isoprene metabolism.

Activity toward the synthetic substrates CDNB/DCNB varies greatly for bacterial GSTs, typically ranging from 0.01 to 10 U mg^−1^. For example, the specific activities of GSTs from Pseudomonas strains range from 0.105 to 1.57 U mg^−1^ (33, 36). WS11-IsoI and WS11-IsoJ displayed activities that are within the lower range for bacterial GSTs. To quantify WS11-IsoI activity when using the natural substrate, the disappearance of epoxyisoprene was followed using GC-MS. In reactions containing 1 mM glutathione and 1 mM epoxyisoprene, epoxyisoprene was consumed at a rate of 0.519 ± 0.036 U mg^−1^ (see Fig. S1 in the supplemental material), almost 20 times the rate detected with CDNB.

### Demonstration of WS11-IsoI activity using HPLC.

**FIG 3:**
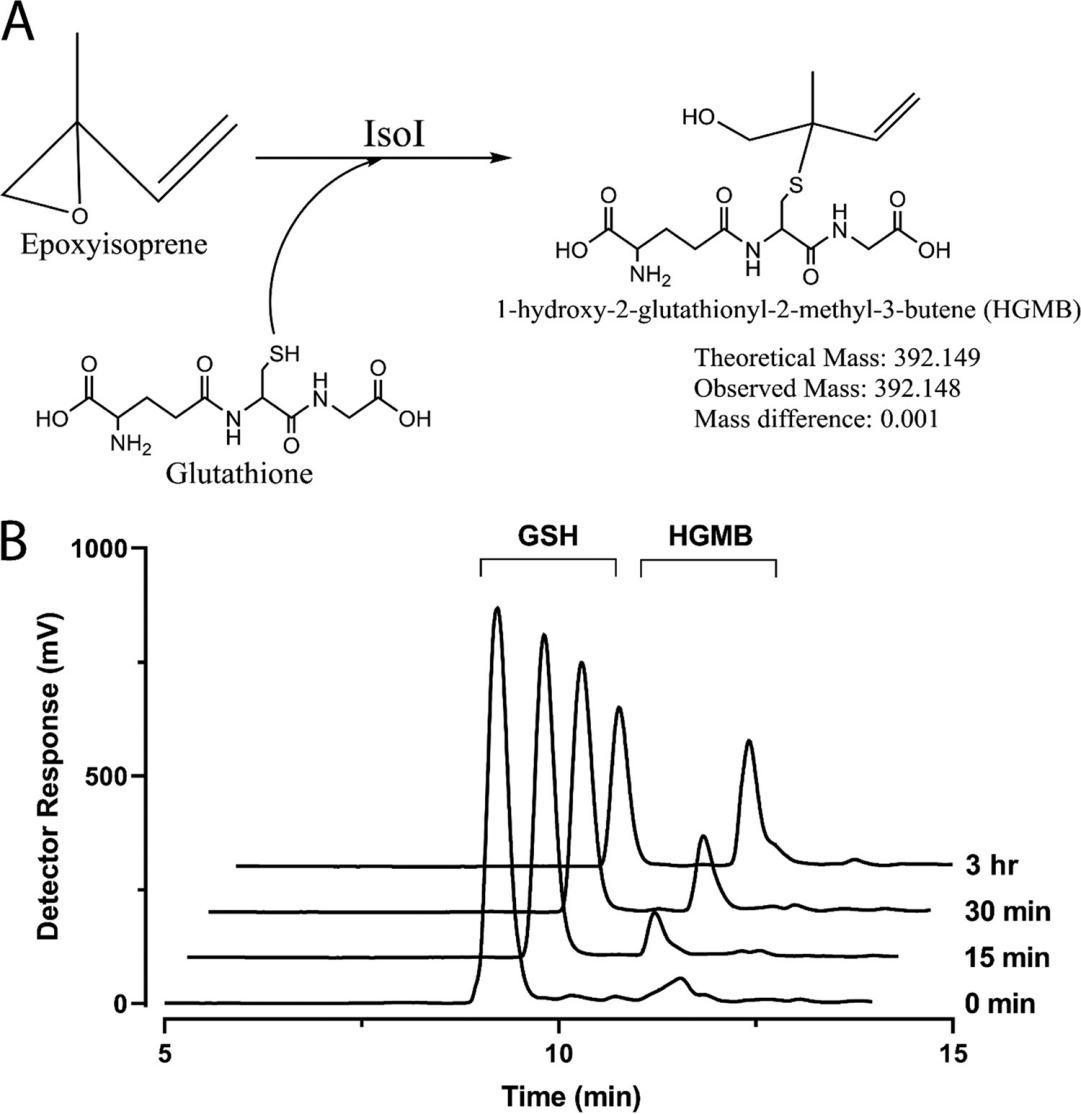
Analysis of the IsoI-catalyzed reaction. (A) Conjugation of glutathione to epoxyisoprene by WS11-IsoI and formation of HGMB with mass confirmation by LC-MS. (B) HPLC chromatograms of the timewise depletion (0 to 3 h) of glutathione and increase in HGMB in samples containing epoxyisoprene and WS11-IsoI.

The activity of WS11-IsoI was also demonstrated by following depletion of glutathione and consequent formation of the glutathione conjugate HGMB, using HPLC. Initially, the disappearance of glutathione (GSH; retention time of ca. 9.1 min) and concomitant formation of a product (retention time of ca. 10.6 min) was observed (Fig. 3). To confirm the identity of this product, the reaction was taken to completion in similar reactions using an excess of WS11-IsoI (Fig. S2). The protein was removed by precipitation, and the sample was concentrated as described in Materials and Methods and analyzed using HPLC to demonstrate the complete conversion of glutathione to HGMB in the solution (Fig. S3). Analysis of the product using LC-MS identified an observed mass of 392.148, consistent with the theoretical value for HGMB (Fig. S4). Also identified was the presence of the protonated dimer, *m/z* 783.291, and other ions showing peptide fragmentation of glutathione, *m/z* 263.108, and for the dealkylated compound with *m/z* 179.043.

### WS11-IsoH dehydrogenase activity with HGMB.

The next step of the isoprene metabolic pathway is the oxidation of the hydroxyl group of HGMB to a carboxylic acid using NAD^+^ as the electron acceptor (27), a reaction assumed to be catalyzed by WS11-IsoH (Fig. 2). Rapid NADH formation was observed by following the increase in absorbance at 340 nm upon addition of NAD^+^ and biosynthesized HGMB to a reaction mixture containing WS11-IsoH. HGMB oxidation followed Michaelis-Menten kinetics, assayed using HGMB from 0 to 5 mM. The *V*_max_ and *K_m_* were 2.96 ± 0.042 U mg^−1^ and 58 ± 3.76 μM, respectively. Specific activity in the presence of NADP^+^ was 0.077 U mg^−1^, less than 3% of that with NAD^+^ (Fig. S5). These data indicate that IsoH from *Variovorax* sp. WS11 has a 40-fold higher affinity for HGMB than IsoH from *Rhodococcus* sp. AD45, which had a *K_m_* of 1.4 mM (27).

The amino acid sequences of WS11-IsoH and AD45-IsoH share 60% identity. Sequence analysis indicated that these enzymes are members of the short-chain dehydrogenase/reductase (SDR) family (27, 37). SDRs constitute a large protein superfamily and encompass a wide variety of enzymes with significant functional diversity (38) involved in detoxification of xenobiotics (39) and lipid, amino acid, and carbohydrate metabolism (40). Despite low sequence identity between members (15 to 30%) these enzymes have conserved structure with characteristic motifs (41). They contain an N-terminal motif, GxxxGxG, that is involved in the binding of the coenzyme NAD^+^ or NADP^+^. Both WS11-IsoH and AD45-IsoH deviate slightly from the classical motif with the substitution of a glycine residue with alanine, GxxxGxA.

### Monitoring WS11-IsoH activity using HPLC.

**FIG 4:**
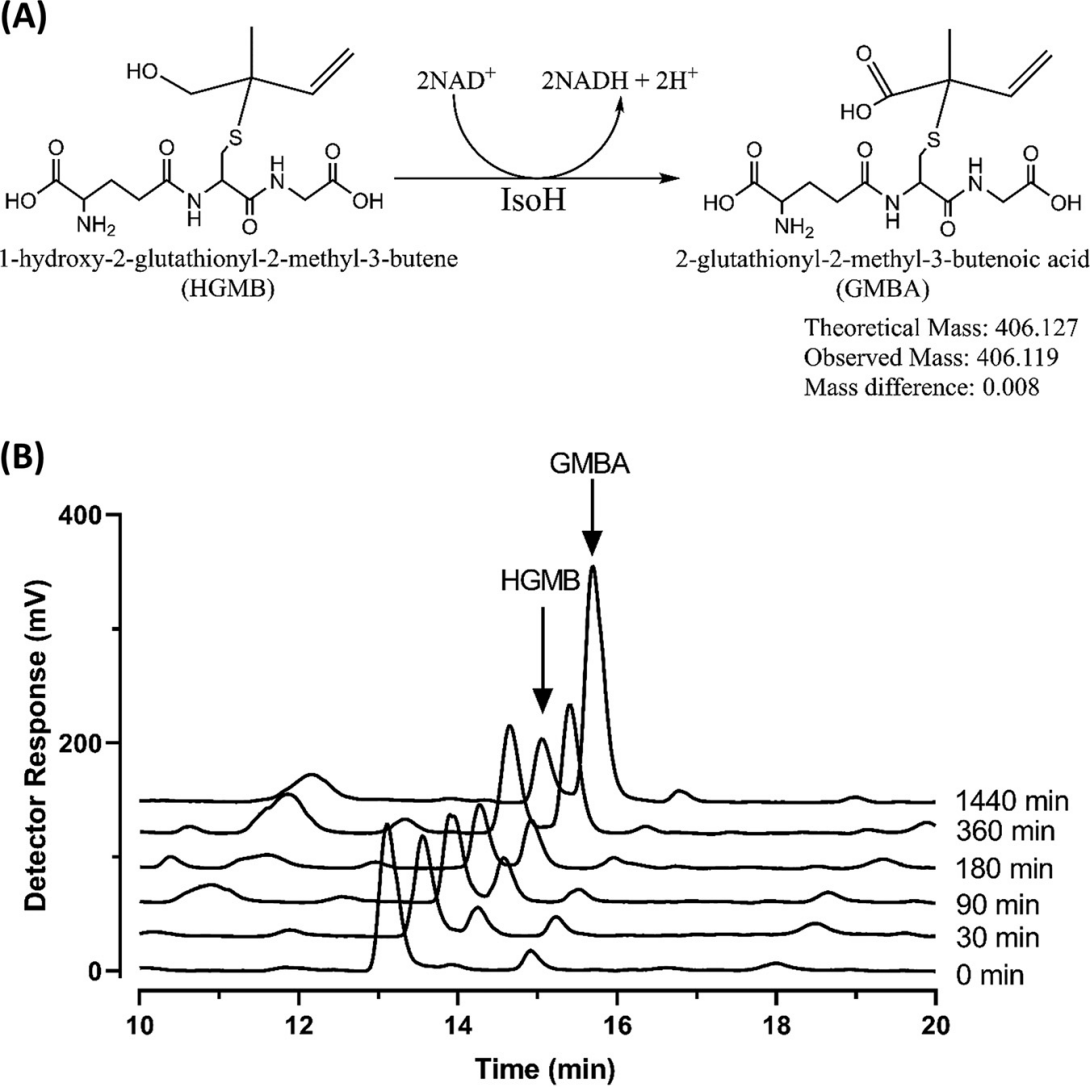
(A) Dehydrogenase action of the two-step enzymatic reaction of WS11-IsoH to produce GMBA from HGMB with mass confirmation by LC-MS. (B) HPLC chromatograms of the timewise depletion (0 to 24 h) of HGMB and increase in GMBA by WS11-IsoH. Samples were taken from a reaction mixture containing 5 mM HGMB and 5 mM NAD^+^ with 0.5 mg mL^−1^ WS11-IsoH, which was incubated at 30°C.

Mixtures of HGMB, NAD^+^, and WS11-IsoH were incubated at 30°C for 24 h, and the formation of product was followed by HPLC (Fig. 4). A modest decrease in HGMB (retention time of ca. 14.6 min) was observed with the formation of a product (retention time of ca. 15.3 min). Analysis by LC-MS showed the presence of a molecular ion with *m/z* 406.119, which is consistent with the value for GMBA (Fig. S6). The intermediate GMB was not detected in any of the samples, suggesting that GMB is immediately further oxidized to GMBA as in *Rhodococcus* sp. AD45 (25, 27). This was also observed during a metabolomics experiment in wild-type *Variovorax* sp. WS11 grown with isoprene and in mutant strains deficient for the essential genes *isoG*, *isoJ*, and *aldH* after incubation with isoprene (42). These experiments illustrate the conserved nature of isoprene metabolism between the Gram-positive *Rhodococcus* sp. AD45 (27) and the Gram-negative *Variovorax* sp. WS11, not only for the initial oxidation of isoprene to epoxyisoprene but also in the subsequent metabolic reactions.

### Comparison of AldH activity from *Variovorax* sp. WS11 and *Rhodococcus* sp. AD45.

**FIG 5:**
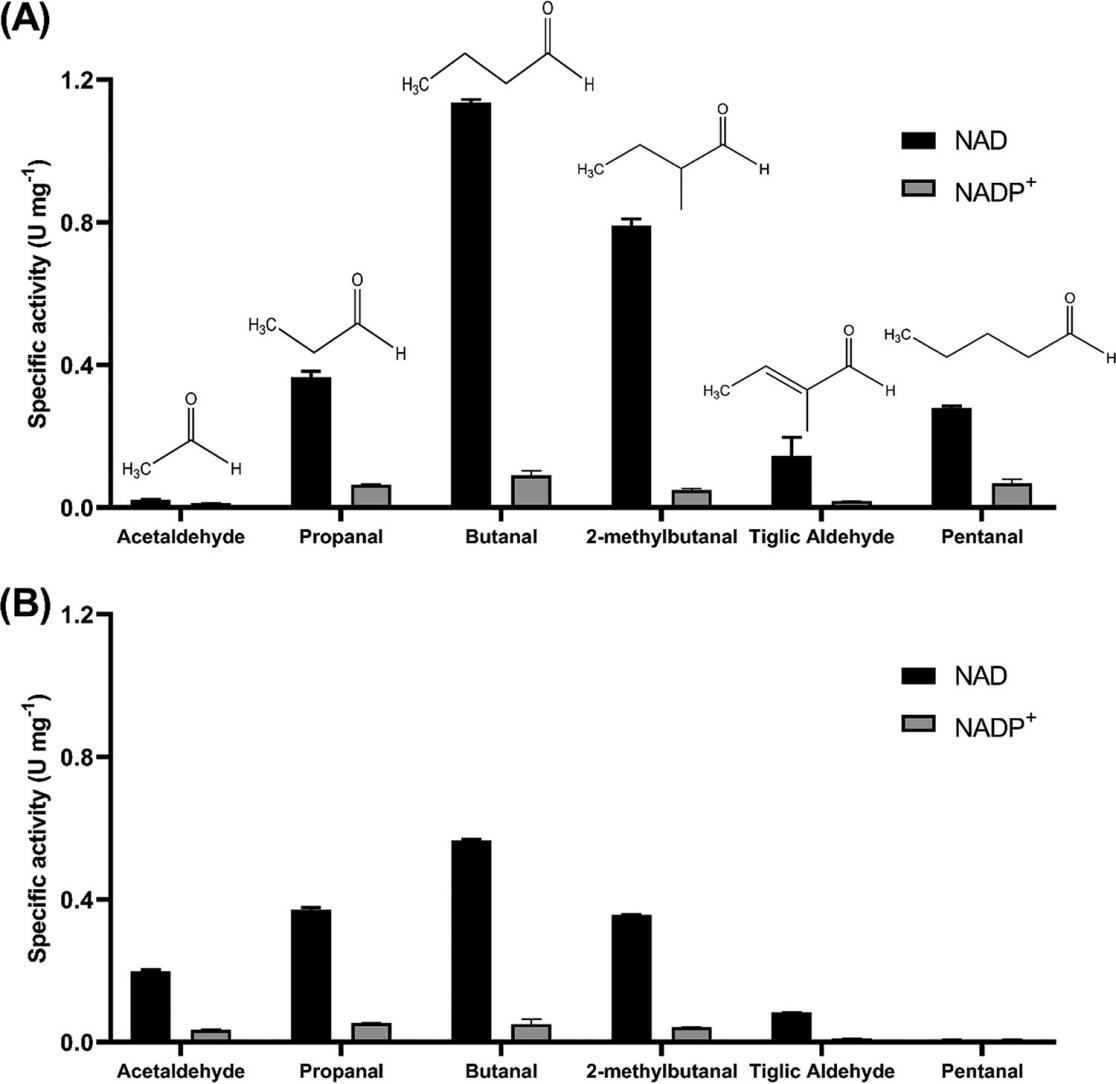
(A and B) The specific activity of (A) WS11-AldH and (B) AD45-AldH with four straight-chain and two branched aldehydes using NAD^+^ or NADP^+^ as the coenzyme. Error bars are standard deviations of the mean of three replicates.

Although the *aldH* gene is conserved in all sequenced *iso* gene clusters (22, 25, 43), no confirmed or even predicted role of AldH has been suggested. However, deletion of *aldH* prevented growth of *Variovorax* sp. WS11 on isoprene (23), thus demanding a more in-depth study of the function of AldH. The substrate specificity of WS11-AldH and AD45-AldH was examined with four straight-chain (C2-C5) and two branched aldehydes (C5), with 2-methylbutanal serving to mimic the isoprene molecule. Glutathione-conjugates of these compounds were not commercially available. Both enzymes and substrates were tested with NAD^+^ or NADP^+^. The location of *aldH* in the *iso* gene clusters of *Rhodococcus* sp. AD45 and *Variovorax* sp. WS11 is shown in Fig. 1. WS11-AldH and AD45-AldH share 52.7% amino acid identity and display a slightly different activity profile and substrate specificity (Fig. 5).

Both enzymes preferred NAD^+^ rather than NADP^+^, with which there was minimal activity. Butanal was the preferred substrate for WS11-AldH and AD45-AldH, with the enzymes reducing NAD^+^ to NADH with activities of 1.14 ± 0.009 and 0.57 ± 0.003 U mg^−1^, respectively. WS11-AldH and AD45-AldH displayed lower activity toward 2-methylbutanal than the unbranched aldehyde, with activities of 0.79 ± 0.019 and 0.36 ± 0.001 U mg^−1^, respectively. Both enzymes displayed identical activities toward propanal of 0.37 ± 0.016 and 0.37 ± 0.006 U mg^−1^ for WS11-AldH and AD45-AldH, respectively. The addition of a carbon-carbon double bond, tiglic aldehyde, caused a drastic decrease in the aldehyde dehydrogenase activity for both enzymes, with an activity of 0.15 ± 0.052 and 0.083 ± 0.0003 U mg^−1^ for WS11-AldH and AD45, respectively. WS11-AldH displayed no activity toward acetaldehyde but was active with pentanal, whereas the reverse was true for AD45-AldH.

Both enzymes displayed the highest *V*_max_ toward butanal, with 1.36 and 0.53 U mg^−1^ for WS11-AldH and AD45-AldH, respectively. Interestingly, AD45-AldH exhibited 8.5 times higher affinity for butanal than WS11-AldH (0.08 versus 0.69 mM). WS11-AldH showed a higher affinity for the branched aldehyde 2-methylbutanal, with a *V*_max_ and *K_m_* of 1.27 U mg^−1^ and 0.12 mM, compared to 0.48 U mg^−1^ and 0.03 mM, respectively for AD45-AldH. Finally, while WS11-AldH was active with propanal, it had a low affinity for the substrate, with a predicted *K_m_* of 10.9 mM; similarly, AD45-AldH had the lowest affinity for this substrate, with a *K_m_* of 0.57 mM.

**FIG 6:**
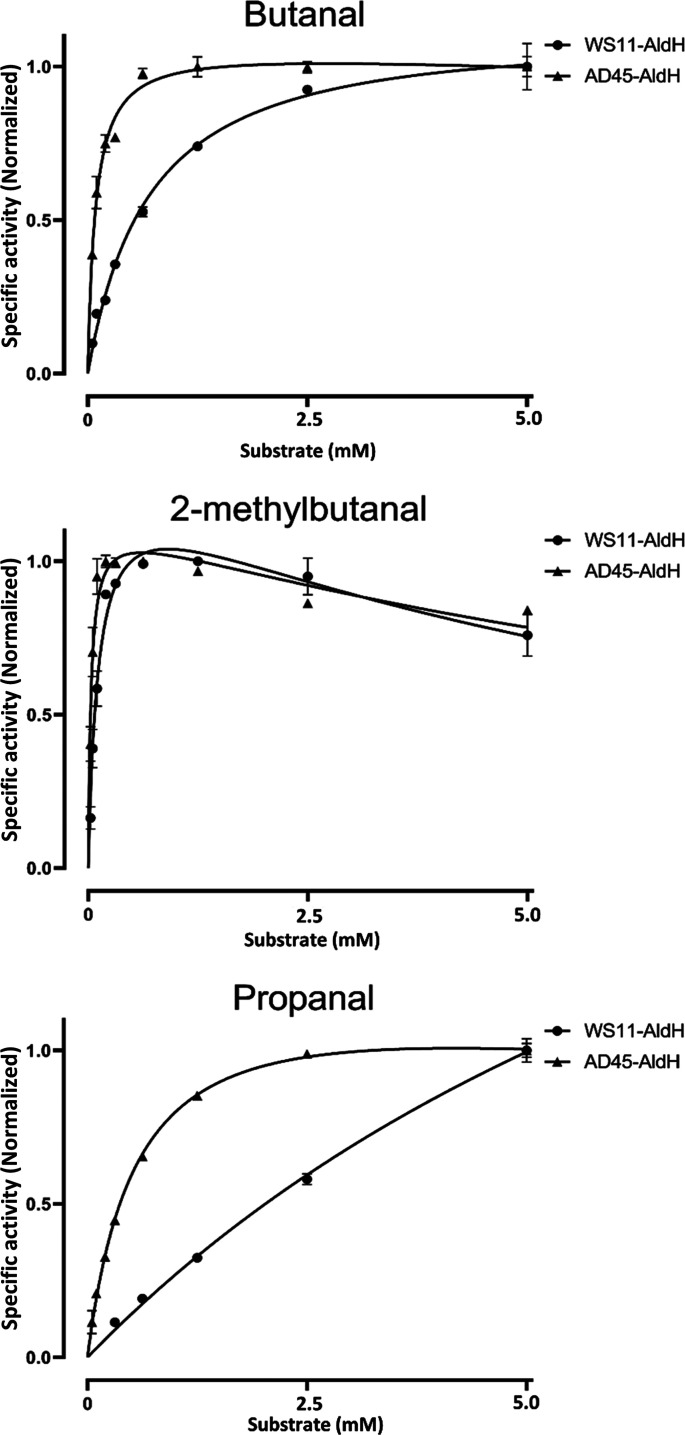
Michaelis-Menten kinetic analysis of WS11-AldH and AD45-AldH with the three most active aldehydes, butanal, 2-methylbutanal, and propanal. Activity was normalized to allow for a comparison of the rate and affinity. The *V*_max_ values for WS11-AldhH were 1,360, 1,269, and 1,050 nmol/min/mg, and the *K_m_* values were 0.69, 0.12, and 10.9 mM, respectively. The *V*_max_ values for AD45-AldH were 527, 484, and 472 nmol/min/mg, and the *K_m_* values were 0.08, 0.03, and 0.57 mM, respectively. Propanal did not saturate WS11-AldH even at 10 mM. Error bars are standard deviations of the mean of three replicates.

A putative role for AldH in the isoprene metabolic pathway is the second oxidation step performed by IsoH, the oxidation of GMB to GMBA (23, 27). To investigate this, the IsoH-catalyzed oxidation of HGMB (assayed as described above) was repeated with the addition of either 0.1 mg/mL WS11-AldH or AD45-AldH, Fig. S7. The addition of WS11- or AD45-AldH more than doubled the rate of conversion of NAD^+^ to NADH, (232% and 277%, respectively). WS11-AldH and AD45-AldH with HGMB alone showed no activity, suggesting that AldH supports IsoH in the conversion of GMB to GMBA (Fig. 2), although it is unclear why such a redundancy would be necessary or, indeed, why AldH should be essential for growth on isoprene (23). Possible explanations include that AldH may catalyze a second essential reaction in the isoprene metabolic pathway, although no such reaction has been proposed, or IsoH may simply be unable to meet the metabolic demand of converting GMB to GMBA and generating reducing power in the form of NADH, necessitating the synergistic activities of IsoH and AldH. Alternatively, although isoprene monooxygenase produces a vast excess of (*R*)-epoxyisoprene (42), some (*S*)-epoxyisoprene is still formed which may accumulate if IsoI cannot form HGMB from this form of the epoxide. AldH may be responsible for the conversion of intermediates derived from (*S*)-epoxyisoprene, thereby potentially preventing toxic accumulations of isoprene pathway intermediates. We now show that AldH has aldehyde dehydrogenase activity (Fig. 5 and 6) and GMB is the only aldehyde intermediate in the proposed isoprene metabolic pathway (23), indicating that the purely synergistic role is more likely.

### Investigating the role of WS11-IsoG.

**FIG 7:**
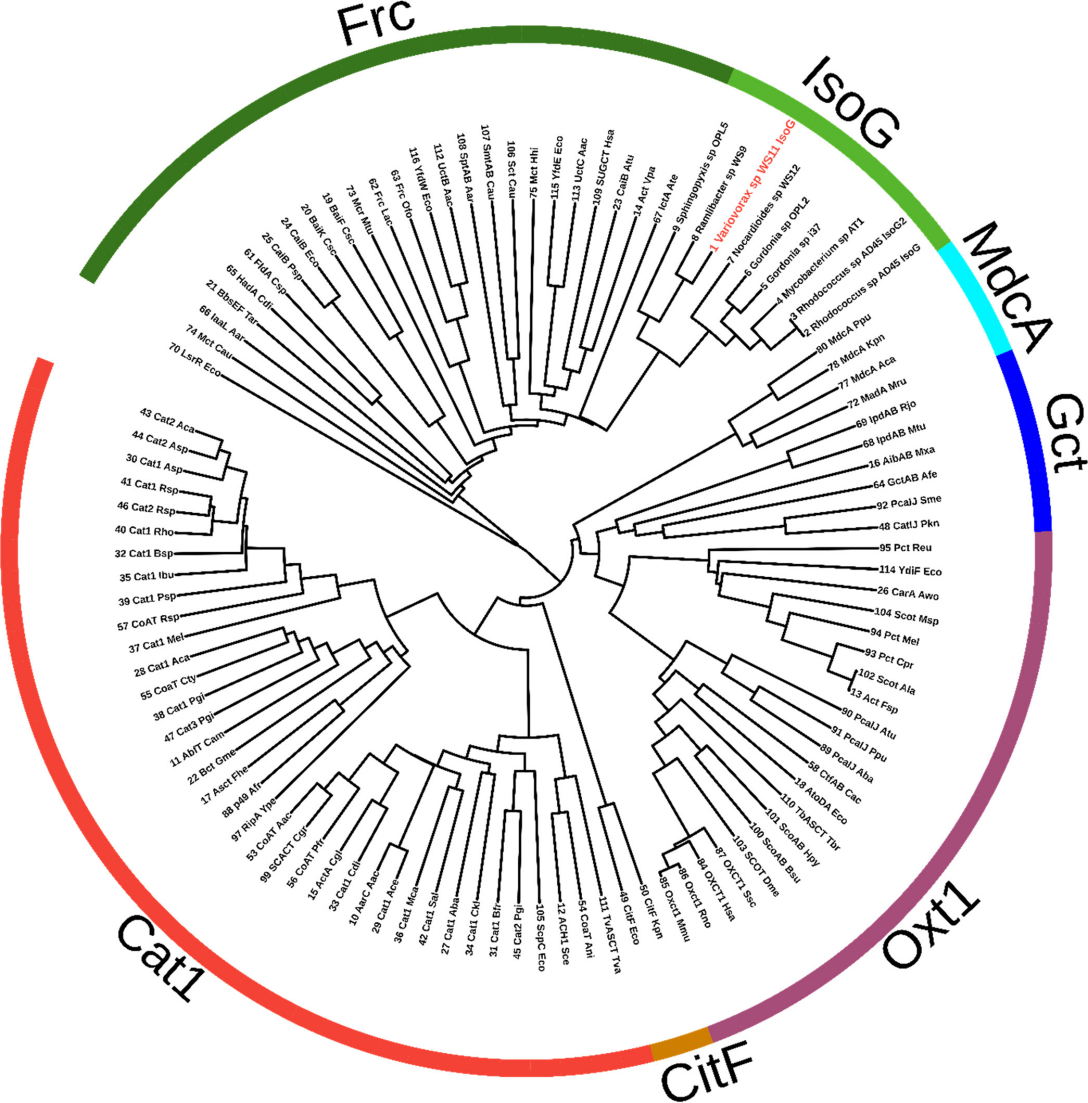
Interactive Tree of Life (iTOL) (45) visualization of 94 published CoA transferases with 9 IsoG sequences from isoprene-degrading bacteria. The CoA transferases separate into six families, Cat1, CitF, Oxt1, Gct, MdcA, and Frc. IsoG sequences cluster within the Frc family.

In *Rhodococcus* sp. AD45, IsoG was originally described as being most similar to an α-methylacyl-coenzyme A racemase from Mus musculus (25). More recent predictions suggest that IsoG acts as a CoA transferase (23, 35) in both *Rhodococcus* sp. AD45 and *Variovorax* sp. WS11. The CoA donor is unknown, preventing detailed characterization of the mechanism of action of this protein. Thus, descriptions of potential activity are based mainly on transcriptomic and proteomic data (23). During growth on isoprene, transcription of *isoG* and expression of IsoG protein were significantly increased compared to uninduced or succinate-grown conditions, and deletion of *isoG* prevented the growth of *Variovorax* sp. WS11 on isoprene (23). Despite this, the role of IsoG remains unconfirmed, although the most logical function is the formation of a GMBA-CoA thioester (23, 25). A detailed bioinformatic analysis was conducted into the role of WS11-IsoG in isoprene metabolism. BLASTp searches of the WS11-IsoG amino acid sequence revealed significant similarity to formyl coenzyme A transferases and carnitine dehydratases. Based on the data set of Hackmann (44), 94 published CoA transferase amino acid sequences with functional information were combined with 9 amino acid sequences of IsoG polypeptides from isoprene-degrading bacteria, and a phylogenetic tree was constructed in iTOL (45) after alignment with MAFFT (46) (Fig. 7).

Earlier studies of the conserved motifs shared by members of the CoA transferase family suggested that three subfamilies existed (47). However, as reported by Hackmann (44), it is more apt to split the CoA transferases into six separate families. IsoG clustered with the formyl CoA transferases, Frc, previously known as the family III CoA transferases (48). Other members include itaconyl-CoA transferase (IctA), carnitine CoA transferase (CaiB), and bile acid CoA transferase (BaiK). (49–51).

AlphaFold 2.1 (52, 53) was used to generate a model of WS11-IsoG (average confidence score of 95.5). The similarity between the predicted structure of WS11-isoG and crystal structures of Frc CoA transferases from E. coli and Oxalobacter formigenes, combined with conservation of the functionally important D169, supports the view that IsoG is a CoA transferase. The architecture of the putative active site of IsoG suggests it could support the reaction. The active site is large enough to accommodate CoA and allow access into the protein core to interact with D169. From this position, several other residues within 3.5 Ä of the thiol moiety of CoA would be available to bond. Of these, residues 16 to 18 are similar to a chain observed in 1T4C and 1PT5, with some substitutions (54). At position 17, 1T4C and 1PT5 have a glutamine which might stabilize tetrahedral intermediates formed between the Asp-CoA complex and CoA-acceptor (54). In IsoG, this is replaced with leucine, a much less reactive residue rarely involved in protein function. Interestingly, CaiB, an (R)-carnitine CoA-transferase, has a similar substitution (I24 > Q17), and while unable to interact with oxalate, it can interact with carnitine (55).

The next steps in the characterization of WS11-IsoG would be to demonstrate CoA transferase activity in an enzymatic assay, requiring the identification of the CoA donor or donors preferred by this enzyme.

### Conclusions.

We report the purification of IsoH, I, and J and AldH from *Variovorax* sp. WS11 and AldH from *Rhodococcus* sp. AD45. WS11-IsoI catalyzes the conjugation of glutathione with epoxyisoprene to form HGMB, and WS11-IsoH was shown to catalyze the two-step oxidation of HGMB to GMBA. Additionally, we demonstrated that AldH could enhance the second oxidation step of HGMB. The exact roles of WS11-IsoJ and WS11-IsoG remain uncertain. However, removal of glutathione in a later step of the metabolic pathway of isoprene was suggested by combined ’omics analyses (23), and glutathione *S*-transferase activity of WS11-IsoJ was demonstrated with the model substrate CDNB. Bioinformatic analysis of WS11-IsoG, performed using a data set of published CoA transferases (44) supports the prediction that WS11-IsoG is a CoA transferase that catalyzes the formation of a GMBA-CoA thioester, which now requires experimental verification.

## MATERIALS AND METHODS

### Cloning of *isoHIJ* and *aldH*.

PCR primers (Table S1) were used to amplify the genes *isoH*, *I*, and *J* and *aldH* from *Variovorax* sp. WS11 genomic DNA (accession no. JAAGOW000000000) using Q5 high-fidelity polymerase (New England Biosciences, USA) according to the manufacturer’s instructions. The *aldH* gene from *Rhodococcus* sp. AD45 (accession no. CM003191) was amplified as described above.

PCR products were purified using a HighPure PCR product purification kit (Roche, Switzerland) and ligated to the pJET1.2/blunt vector using the CloneJET kit (Thermo Fisher, USA), according to the manufacturers’ instructions. The resulting mixture was used to transform E. coli TOP10 chemically competent cells (Invitrogen, USA) according to the manufacturer’s instructions and then plated on LB medium containing ampicillin (100 μg mL^−1^). A GeneJET plasmid miniprep kit (Thermo Scientific, USA) was used to extract plasmid from transformants, and the gene of interest was excised using the appropriate restriction enzymes (Table S1). Aliquots of the target vector (pET16b [Novagen, UK] for expression of WS11-IsoH, WS11-IsoI, WS11-AldH, AD45-AldH), and pET20MBP (a modified pET20 vector [Novagen, UK], which allows for N-terminal fusion of maltose binding proteins to enhance solubility of heterologous proteins, generously gifted by Nick Burton) for expression of WS11- IsoJ were digested with the appropriate restriction enzymes (Table S1). Digested insert and vector were extracted from agarose gels, purified using a HighPure PCR product purification kit (Roche), and ligated using T4 DNA ligase (Thermo Fisher), and the resulting ligation mix was used to transform E. coli TOP10 cells, which were then plated on LB medium containing ampicillin (as described above). Plasmid minipreps were prepared as outlined above, and plasmid sequences were verified.

### Recombinant protein expression and cell lysate preparation.

Chemically competent E. coli Rosetta2 (pLysS) (Sigma-Aldrich, USA) cells were transformed with pET16b:WS*isoH*, pET16b:WS*isoI*, pET16b:WS*aldH*, pET16b:AD*aldH*, and pET20MBP:WS*isoJ*, (Table S2) according to the manufacturer’s instructions and then plated on LB agar containing both ampicillin (as described above) and chloramphenicol (35 μg mL^−1^). Transformants were used to inoculate 500 mL LB medium containing ampicillin and chloramphenicol (as described above) and were grown at 37°C to an optical density at 540 nm (OD_540_) of 0.6 to 0.8, followed by induction with 75 μM IPTG (isopropyl-β-d-thiogalactopyranoside). The cultures were then grown at 30°C overnight before harvesting by centrifugation (8,000 × *g*, 4°C, 20 min). Cell pellets were resuspended in the relevant buffer, outlined below, for the first step of the purification process, snap-frozen in liquid nitrogen, and stored at –80°C until use.

### Purification of His-tagged isoprene metabolic enzymes.

Protein purification was performed using an ÄKTA pure protein purification system (Cytiva, USA). Cell pellets containing WS11-IsoH, WS11-IsoI, WS11-AldH, and AD45-AldH were thawed and resuspended in 10 mL buffer containing 50 mM Tris-HCl and 30 mM imidazole (pH 9.0) and broken by sonication (15 s on, 45 s off) for a total of 10 min. Cell debris was removed by centrifugation (16,000 × *g*, 4°C, 45 min), followed by 0.45-μm syringe filtration (Sartorius, Germany), and the resulting cell extract was applied to a 5-mL HisTrap fast-flow (FF) column (Cytiva). Elution of bound protein was achieved using a 50-mL gradient from 30 mM to 500 mM imidazole. Fractions containing the protein of interest (as determined by SDS-PAGE) were pooled. Glycerol was added to WS11-IsoI samples (20% vol/vol) to prevent precipitation upon thawing, and all purified proteins were snap-frozen in liquid nitrogen in 1-mL aliquots for subsequent characterization.

### Purification of MBP-fusion enzymes.

Cell pellets containing WS11-IsoJ (expressed with N-terminal maltose binding protein [MBP] fusions from pET20MBP:WS*isoJ*) were thawed and resuspended in buffer containing 50 mM Tris-HCl and 50 mM NaCl (pH 7.4) and broken as described above. Cell extract was applied to a Q Sepharose column (Cytiva, USA; bed volume, approximately 130 mL), preequilibrated in the same buffer. The column was washed in the same buffer, followed by elution using 1 M NaCl. Fractions were checked for the presence of the proteins of interest by SDS-PAGE, and WS11-IsoJ was found in the flowthrough and wash fractions. These fractions were pooled and concentrated to a final volume of 20 mL using a Corning Spin-X ultrafiltration (UF) centrifugal concentrator which applied a 5-kDa cutoff. The concentrated sample was dialyzed into buffer containing 20 mM Tris-HCl and 200 mM NaCl (pH 7.4) and applied to a 5-mL MBPTrap high-performance (HP) column (Cytiva) preequilibrated in the same buffer, and then the column was washed with a further 25 mL of buffer. Proteins were eluted in a single step by the addition of 10 mM maltose, and the resulting purified protein sample was further concentrated to 2.5 mL (as described above). Finally, this concentrated protein sample was applied to a Superdex HiLoad 26/600 200-pg-size exclusion column (Cytiva) equilibrated in 50 mM phosphate buffer and 150 mM NaCl (pH 7.0). Aliquots of purified protein were snap-frozen with liquid nitrogen and stored at –80°C.

### Strains and plasmids.

Strain and plasmid information can be found in Tables S2 and S3.

### WS11-IsoI glutathione *S*-transferase assay.

The activity of recombinant WS11-IsoI was assayed spectroscopically in a preheated Shimadzu UV-1800 spectrophotometer with a Shimadzu Constant-Temperature Measurement (CPS)-controller using 1-chloro-2,4-dinitrobenzene (CDNB) (340 nm) and 1,2-dichloro-4-nitrobenzene (DCNB) (345 nm) as adapted from Lienkamp et al. (31) and van Hylckama Vlieg et al., (25). Briefly, the reactions contained 50 mM potassium phosphate buffer (pH 7.0), 10 mM glutathione, 0.5 mg mL^−1^ recombinant WS11-IsoI, and 1.5 mM CDNB/DCNB in a final volume of 1 mL in a quartz cuvette and were performed at 30°C. Due to a high abiotic reaction rate at higher pH values, pH 7.0 was selected for these assays.

The reaction mixture was incubated for 2 min at 30°C before either CDNB or DCNB was added to initiate the reaction. Activities were calculated using extinction coefficients of 8.5 and 9.6 mM^−1^ cm^−1^ for DCNB and CDNB conjugates, respectively (56, 57), and were corrected for the abiotic reaction rate.

### Preparation of HGMB samples for HPLC.

WS11-IsoI glutathione *S*-transferase activity was analyzed by HPLC. Briefly, the 1-mL reaction mixture contained 50 mM sodium carbonate buffer (pH 10.0), 0.5 mg mL^−1^ WS11-IsoI, 5 mM glutathione, and 5 mM epoxyisoprene. The reaction mixture was incubated at 30°C, and samples were taken over a period of 3 h for HPLC analysis. The protein was precipitated from 100-μL aliquots of reaction mixture by adding 2 μL of 100% trichloroacetic acid (TCA). Samples were incubated for 15 min at 4°C before being centrifuged (21,000 × *g*, 4°C, 15 min). The supernatant was removed and added to a fresh 1.5-mL centrifuge tube. The sample was then appropriately diluted in distilled water (dH_2_O) to make up to a 750-μL volume for HPLC assays.

### Identification of glutathione, HGMB, and GMBA by HPLC.

HPLC methods were developed to determine the levels of glutathione, HGMB (1-hydroxy-2-glutathionyl-2-methyl-3-butene), and GMBA (2-glutathionyl-2-methyl-3-butenoate) as fluorescent adducts after precolumn derivatization with ortho-phthalaldehyde (OPA; Sigma-Aldrich, P0657), using β-mercaptoethanol. Samples (750 μL; preparation described above) were mixed in a 1:1 ratio with derivatization reagent (10 mg OPA in 1 mL methanol, buffered with 7 mL 1 M potassium borate buffer [pH 10.4], mixed with 16 μL β-mercaptoethanol) before injecting a 20-μL sample onto a Synergi 4-μM Hydro-RP 80-Å 100 by 2-mm column (Phenomenex, USA) and eluting at 0.35 mL min^−1^. A gradient was delivered from buffer reservoirs: A (25 mM sodium phosphate [pH 2.5]) and B (methanol/acetonitrile/water at a ratio of 5:4:1 [vol/vol/vol]) using the following gradient: percentage of B, 10 to 80% from 0 to 12 min. The duration of the gradient was extended further to separate HGMB and GMBA, from 0 to 23 min. A Dionex RF 2000 fluorescence detector was used to detect the products with excitation at 330 nm and emission at 450 nm.

### Purification of HGMB.

Purified WS11-IsoI was used to synthesize HGMB using 20 mM glutathione and 25 mM epoxyisoprene in a 5-mL reaction mixture containing 50 mM sodium carbonate buffer (pH 9.0). The reaction was initiated by the addition of WS11-IsoI to 1 mg mL^−1^, and the mixture was incubated for 3 h at 30°C. Samples (preparation described above [100 μL]) were removed from the reaction mixture and analyzed by HPLC to confirm that the reaction had completed and all glutathione had been consumed. The reaction mixture was filtered using a Spin-X filter (Corning), as detailed previously, to remove WS11-IsoI. The filtrate was lyophilized to remove residual epoxyisoprene, and the sample was resuspended in 200 μL dH_2_O to an estimated concentration of 0.5 M, predicted according to the initial concentration of glutathione. Additional samples were taken and analyzed by LC-MS.

### WS11-IsoH dehydrogenase assays.

WS11-IsoH dehydrogenase activity was assayed by following the change in absorbance at 340 nm associated with the formation of NADH from NAD^+^. The 1-mL reaction mixture, prepared in a quartz cuvette, contained 50 mM sodium carbonate buffer (pH 10.0), 0.02 mg WS11-IsoH, and 5 mM HGMB and was incubated for 2 min at 30°C in a preheated Shimadzu UV-1800 spectrophotometer with a Shimadzu CPS-controller. The reaction was initiated by the addition of 5 mM NAD^+^, and the cuvette was mixed by inversion. The change in absorbance at 340 nm was measured for 5 min.

WS11-IsoH dehydrogenase kinetics activity was assayed over 14 substrate concentrations in the range of 5 mM to 5 μM HGMB in 50 mM sodium carbonate (pH 10.0) for 2 min. Specific activity is reported as U mg^−1^, where one unit (U) is defined as the amount of enzyme that catalyzes the conversion of one micromole of substrate per minute under the assay conditions. Michaelis-Menten parameters were calculated using GraphPad Prism 8.0.1.

### WS11-AldH and AD45-AldH substrate specificity and kinetics.

The activity of recombinant WS11-AldH and AD45-AldH was assayed spectroscopically by following the change in absorbance at 340 nm associated with the formation of NAD(P)H from NAD(P)^+^. The 1-mL reaction mixture, prepared in a quartz cuvette, contained 50 mM sodium carbonate buffer (pH 10.0), 0.1 mg WS11-AldH or AD45-AldH, and 5 mM substrate (acetaldehyde [Sigma; W200379], propanal [Thermo Fisher; 131510250], butanal [Sigma; 8.01555], 2-methyl butanal [Sigma; M33476], tiglic aldehyde [Sigma; 192619], and pentanal [Sigma; 110132]). All chiral aldehydes purchased for use in this study were chemically synthesized and therefore were racemic mixtures. The reaction was initiated by the addition of 5 mM NAD^+^/NADP^+^, and the cuvette was mixed by inversion. The change in absorbance at 340 was measured for 5 min.

WS11-AldH and AD45-AldH were assayed at substrate concentrations in the range of 10 mM to 25 µM for the three fastest substrates, butanal, 2-methylbutanal, and propanal. Michaelis-Menten parameters were calculated using GraphPad Prism 8.0.1. For 2-methylbutanal, the substrate inhibition model was used at the high concentrations, as the substrate began to inhibit activity.

WS11-IsoH dehydrogenase assays (described above), using 1 mM HGMB, were performed with the addition of 0.1 mg/mL WS11-AldH or AD45-AldH to determine if the rate of the dehydrogenase activity increases.

### Preparation of GMBA samples.

WS11-IsoH dehydrogenase activity was analyzed by HPLC. The 1-mL reaction mixture, prepared in a 2-mL screw-cap vial (Agilent Technologies, USA), contained 50 mM sodium carbonate buffer (pH 10.0), 0.02 mg WS11-IsoH, and 5 mM HGMB. The reaction mixture was incubated at 30°C, and samples were taken over a period of 24 h. Samples (preparation described above) were analyzed using HPLC and LC-MS.

### LC-MS analysis of HGMB and GMBA.

LC-MS analyses of HGMB and GMBA samples were conducted by the UEA Sci-Analytical Facility (University of East Anglia, Norwich, UK). Samples were run in positive-ion mode. For data processing, the analytical data processing software from ACD/Labs 2021.2.2 (Toronto, Canada) was used.

### Investigation of epoxyisoprene depletion by WS11-IsoI using GC-MS analysis.

Reaction mixtures (1 mL) containing 2 mM epoxyisoprene, 2 mM GSH, and 0.1 mg mL^−1^ WS11-IsoI in 50 mM sodium carbonate (pH 9.0) were incubated for 10 min at 30°C. Samples were taken at the *T*0 min and *T*10 min time points, and solvent was extracted using diethyl ether. To 0.6 mL diethyl ether, 0.4 mL of the reaction mixture was added, and the mixture was vortexed vigorously for 2 min. The mixture was then centrifuged (16,200 × *g*, 4°C, 1 min) before being incubated in ice for 2 min. The upper, organic layer was removed and placed in a 250-μL vial for analysis by GC-MS.

### Quantification of epoxyisoprene using GC-MS.

Epoxyisoprene in samples was quantified by GC-MS, using a GCMS-QP2010s instrument (Shimadzu, UK) fitted with an AOC-20s autosampler. Helium was used as a carrier gas at 0.9-mL min^−1^ column flow (VF-624ms, 30 m by 0.25 mm, 1.4 μm film; Agilent Technologies, UK). The inlet temperature was 250°C, and the oven was maintained at 50°C for 3 min, ramped to 80°C at 15°C min^−1^, held for 1.33 min, ramped to 160°C at 30°C min^−1^, and held for 2 min. The interface and ion source were at 250°C and 200°C respectively. Samples (2 μL) were injected in split mode (1:20 split). Initially, mass spectra were obtained by scanning from *m/z* 35 to 500 and comparison with the National Institute of Standards and Technology Mass Spectral Database (NIST 08). The retention time for epoxy isoprene was 5.48 min. Epoxyisoprene was quantified in selected ion monitoring (SIM) mode, targeting ions 39, 43, and 53 (*m/z*) and using GCMS solution v.2.50 (Shimadzu), against a calibration curve consisting of six dilutions of commercially available epoxy isoprene (0.002 to 4 mM) (Merck, UK).

### Bioinformatics analysis of WS11-IsoG.

The coenzyme A transferase data set was downloaded from Hackmann (44) and supplemented with IsoG amino acid sequences from known isoprene degraders. This data set was aligned using MAFFT (46) using the auto strategy for iterative refinement methods. A phylogenetic tree of the aligned amino acid sequences was produced using the average linkage (UPGMA) method built into MAFFT. The phylogenetic tree was then visualized using iTOL (45).

A multisequence FASTA file containing two copies of the amino acid sequence of IsoG from *Variovorax* sp. WS11 was used as the template for AlphaFold (version 2.1.1) (53) predictions, due to the likelihood that WS11-IsoG exists as a dimer, as determined by its predicted status as an Frc-CoA-like transferase (52, 53). The predictions were carried out on a high-performance computing node with an Intel Xeon Silver 4116 CPU, NVIDIA Quadro P5000 2.1-GHz graphics processing unit (GPU), and 60 GB RAM. Multiple sequence alignments were built using the full set of genetic databases, and the maximum template date was set to 14 May 2021. AlphaFold requires the following genetic databases to build the multiple-sequence alignment (MSA) and hidden Markov model for the prediction: BFD, MGnify, PDB, Uniclust30, UniRef90, UniProt, PDB seqres. A dimeric model was chosen because other members of the Frc family, including Oxalobacter formigenes (PDB 1T4C) and Escherichia coli (PDB 1PT5) (54, 58), were crystalized in a dimeric form. All images were generated using PyMOL version 2.5.1 (59).
