# Supplementary material for: Analysis of Essential Isoprene Metabolic Pathway Proteins in Variovorax sp. Strain WS11
Source: Appl Environ Microbiol. 2023 Feb 22;89(3):e02122-22. doi: 10.1128/aem.02122-22 (PMC10057887; doi:10.1128/aem.02122-22)
Supplement: Supplemental file 1 — Supplemental material. Download aem.02122-22-s0001.pdf, PDF file, 0.4 MB [file aem.02122-22-s0001.pdf]

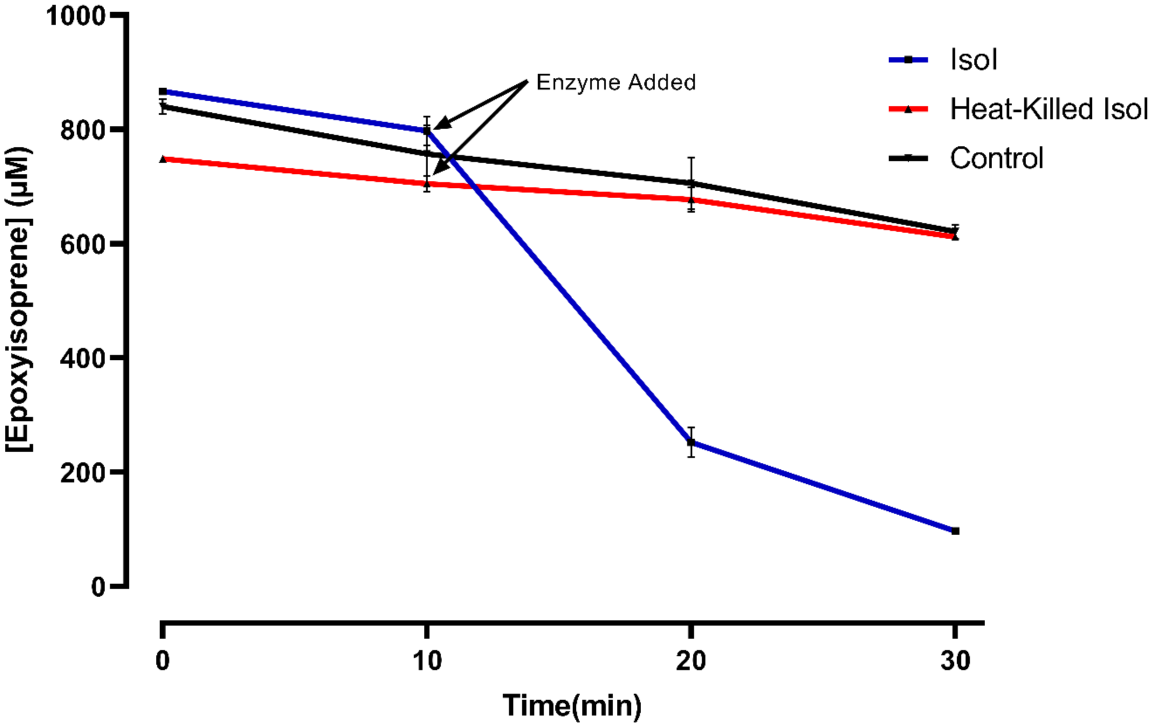

Supplementary Figure 1. Depletion of epoxyisoprene after the addition of 0.1 mg mL^{-1} IsoI or heat-killed IsoI, measured by GC-MS. Reactions where enzyme was added at 10 min are shown together with the non-enzyme control. Error bars are standard deviations about the mean of three replicates.

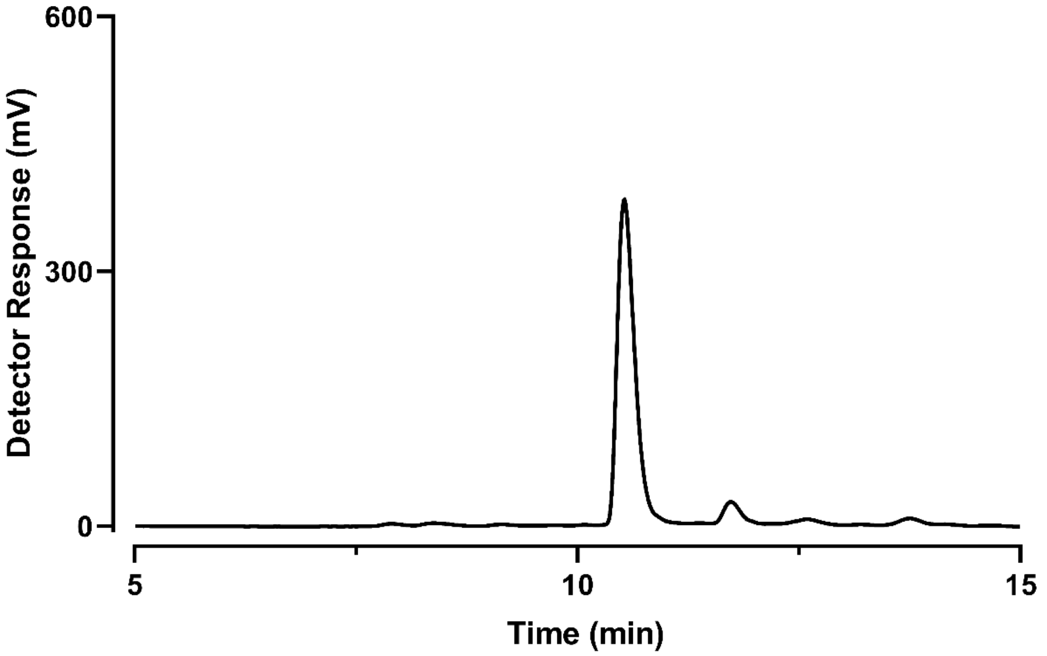

Supplementary Figure 2. The complete conversion of 20 mM glutathione after incubation with 1 mg mL^{-1} IsoI and 25 mM epoxyisoprene for three hours at 30 °C.

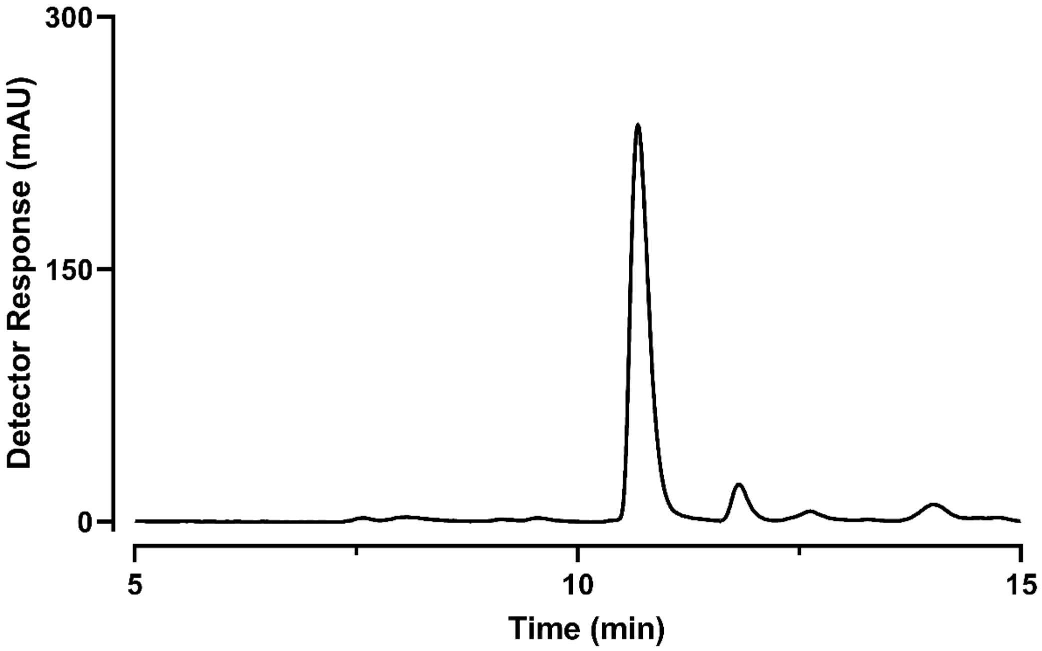

Supplementary Figure 3. Purity of freeze-dried HGMB. Approximately 1 µL of 500 mM HGMB in a 1.5 mL HPLC sample.

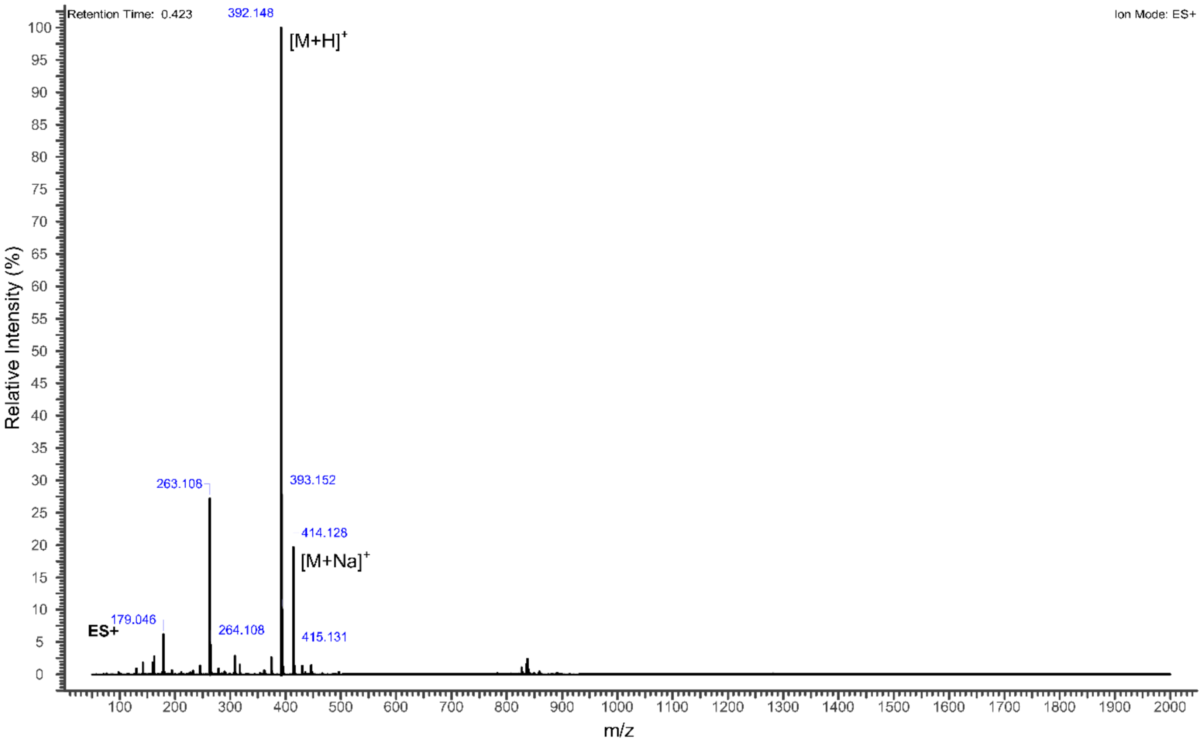

Supplementary Figure 4. LC-MS mass spectrum of enzymatically produced HGMB.

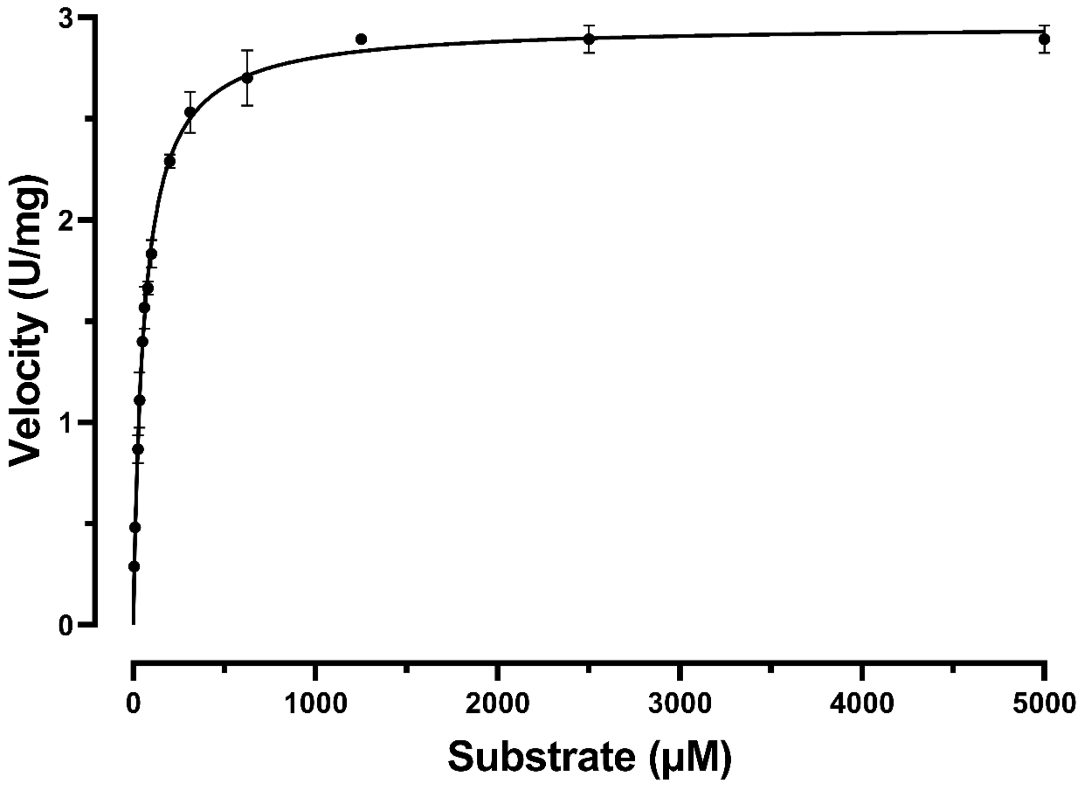

Supplementary Figure 5. Michaelis-Menten kinetics of WS11-IsoH towards HGMB, R^2=0.9944. The V_{max} and K_m values were 2.96 ± 0.042 U mg^{-1} and 58 ± 3.76 µM respectively. Error bars are standard deviations about the mean of three replicates.

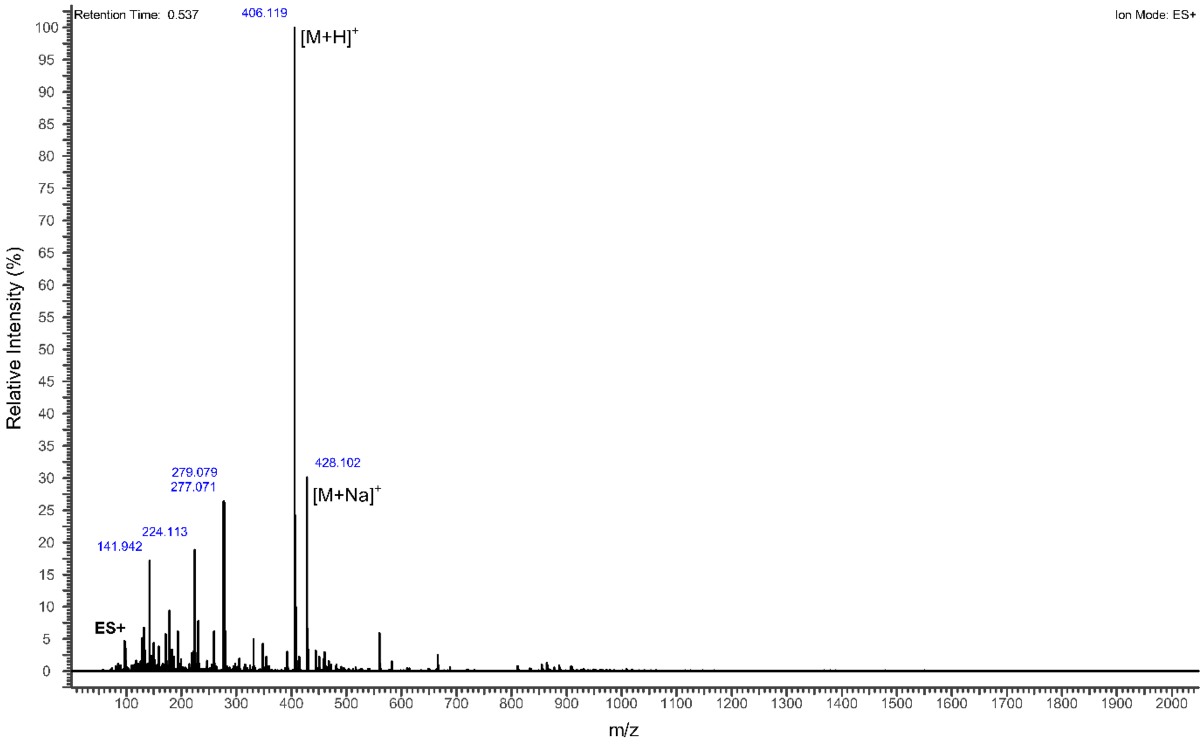

Supplementary Figure 6. LC-MS mass spectrum of enzymatically produced GMBA.

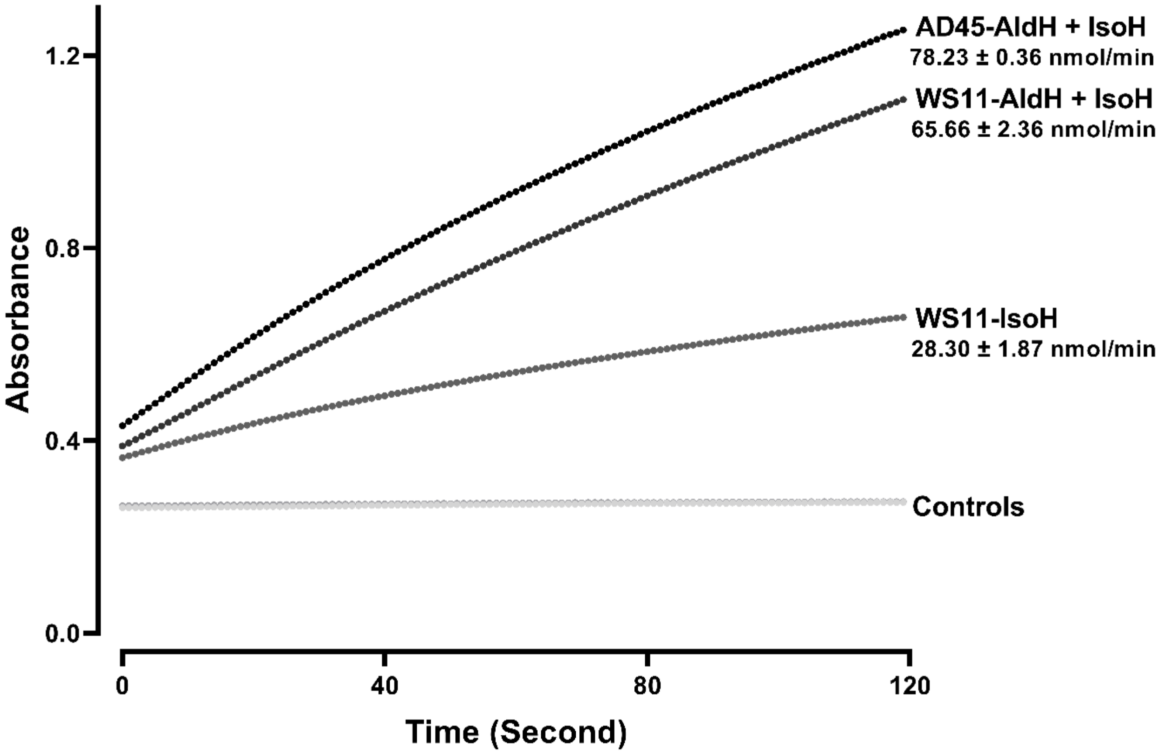

Supplementary Figure 7. Examining the role of AldH in the third and fourth step of the isoprene metabolic pathway, production of GMB and GMBA, through comparison of the rate of NADH formation through change in absorbance. Additions of both AD45-AldH and WS11-AldH to a reaction mixture containing WS11-IsoH and HGMB show a large increase in the rate of NADH formation. Controls of WS11-AldH and AD45-AldH without WS11-IsoH were performed to demonstrate that AldH is not involved in the third step of the pathway.

```
WS11-IsoG.       --RPRLI-RTV-LPDKLAA-VTVAQSISAALVSKLSTG
Ramlibacter.sp.  --RPRLI-RTV-LPDKLAA-VTVAQSLSAALVSRHRTG
Sphingopyxis.sp  --RPRLI-RTV-LPDKLAA-VMAAQTMTAALVGRGTEG
Rhodococcus.sp.  --RPRLI-RTV-LPDKLSA-VFAAQAITAALFARERTG
Rhodococcus.sp.  --RPRLI-RTI-LPDKLSA-VFAAQAITAALFARERTG
Mycobacterium.s  --RPRLI-RTI-LPDKLTA-VVAAQTLSAALFARERTG
Gordonia.sp.i37  --RPRLI-RTI-LPDKLTA-VVAAQALSAALFARERSG
Gordonia.sp.OPL  --RPRLI-RTI-LPDKLTA-VVAAQALSAALFARERSG
Nocardioides.sp  --RPRLI-RTI-LPDKLSA-VVGAQAISAALFARERNG
CaiB.Atu         ---PMKA-GVA-VADIFTG-IYSVSAIQAALIHAMRSG
Act.Vpa          ---PMKT-GVG-IADVMTG-MYAAVGILAALQHRNVTG
SUGCT.Hsa       ----PVRP-GVA-MTDLATG-LYAYGAIMAGLIQKYKTG
UctC.Aac         ---PTRVGGTS-LSDLCGG-VFMFCGIASALYARERTG
YfdE.Eco         ---PVRV-GTS-LADLCGG-VYLFSGIVSALYGREKSQ
Mct.Hhi          ---PTKV-GPG-VGDLFTA-VLNAVGILAAVHHRERTG
Sct.Cau          ---PTRI-GIS-IGDSLAA-TFAALGALVALHQRQRSG
SmtAB.Cau        ---PVLP-GTAPLGDYIAS-LFGAIGILIALRHKEQTG
Frc.Lac          FNVPTQS-AAA-LGDSNSG-MHLTIAILAALMQREHTG
Frc.Ofo          ---PTVS-GAA-LGDSNSG-MHLMIGILAALEMRHKTG
UctB.Aac         NNGPLVS-AAA-LGDSNTG-NHLLIGVLAALFGRERTG
YfdW.Eco         ---PLVS-AAA-LGDSNTG-MHLLIGLLAALLHREKTG
SptAB.Aar       ----PMLL-YGP-YIDFIAS-TLGASAVLAALDRQRRIG
IctA.Ate         ---PAKV-GIS-IADISAG-CYAYSNILAALIQRDKDP
CaiB.Eco         ---PMPA-FPY-TADYFSG-LTATTAALAALHKARETG
CaiB.Psp         ---PMPA-FPY-TADYFSG-MTATTSALAALYKVQQTG
BaiF.Csc         ---ALKI-NPY-LSDFVCG-LTTCWAMLACYVSTILTG
BaiK.Csc         ---AMKV-SPY-LSDYVTA-LNTCWTALAAYVHVLRTG
HadA.Cdi         --SPANT-AAG-FGDHYAG-LALAAGSLAALHKKAQTG
Mcr.Mtu          ---PVPP-LNL-VGDFGGGSMFLLVGILAALWERQSSG
IaaL.Aar         ---PTLA-GVP-IVDLKAG-DEVFANVMLALLERAETG
Mct.Cau          ---DVVN-HVLPAWDIVTG-QMIALGLLAAERHRRLTG
                              *    .
```

Figure S8. Sequence alignment of a portion of the active site region of Frc CoA transferases with the conserved Aspartate residue highlighted in yellow.

Supplementary Table 1 – PCR Primers used to amplify IsoH, IsoI and IsoJ from *Variovorax* sp. WS11 for ligation into pJET1.2. All forward primers contained a *Nde*I and reverse primers a *Bam*HI restriction site (shown in italics).

WS11-*isoH*	F 5'- atatat*catATG*ACGAAGAACATTCTGGTG-3'<br>R 5'- atatat*ggatcc*CTACCAGAGCAACTGTTCC-3'
WS11-*isoI*	F 5'- atatat*catATG*ATCACTCTTTACCAATACATGCC-3'<br>R 5'- atatat*ggatcc*TCAGATGCCGAAGCGCT-3'
WS11-*isoJ*	F 5'- atatat*catATG*ATCGAACTCTACTACTACACC-3'<br>R 5'- atatat*ggatcc*TCAGTTGCTGATGGATGTGG-3'

Supplementary Table 2 – List and brief description of the plasmids used to clone and purify IsoHIJ.

Plasmids	Description	Reference/Source
pJETblunt	Amp^R cloning vector	Thermofisher Scientific
pET16b	Amp^R expression vector for N-terminal His-tag	Novagen/Invitrogen
pET20MBP	Amp^R expression vector for N-terminal MBP fusion	Modified pET20 (Novagen) vector, gifted by Dr Nicolas Burton
pET16b:WS*isoH*	pET16b containing *isoH* from *Variovorax* sp. WS11	This study
pET16b:WS*isoI*	pET16b containing *isoI* from *Variovorax* sp. WS11	This study
pET20MBP:WS*isoJ*	pET20MBP containing *isoJ* from *Variovorax* sp. WS11	This study

Supplementary Table 3 – List and brief description of the strains used to clone and purify IsoHIJ.

Strains	Description	Reference/Source
Escherichia coli TOP10	Strain used for cloning and plasmid maintenance	Invitrogen
Escherichia coli Rosetta2 (pLysS)	Strain used for protein expression. Chl^R	Novagen
